# Thinning of originally-existing, mature myelin represents a nondestructive form of myelin loss in the adult CNS

**DOI:** 10.3389/fncel.2025.1565913

**Published:** 2025-03-11

**Authors:** Min Li Lin, Wensheng Lin

**Affiliations:** ^1^Department of Neuroscience, University of Minnesota, Minneapolis, MN, United States; ^2^Institute for Translational Neuroscience, University of Minnesota, Minneapolis, MN, United States

**Keywords:** myelin, myelin thickness, myelin thinning, myelin loss, myelin disorder, oligodendrocyte, PERK

## Abstract

The main function of oligodendrocytes is to assemble and maintain myelin that wraps and insulates axons in the central nervous system (CNS). Traditionally, myelin structure, particularly its thickness, was believed to remain remarkably stable in adulthood (including early and middle adulthood, but not late adulthood or aging). However, emerging evidence reveals that the thickness of originally-existing, mature myelin (OEM) can undergo dynamic changes in the adult CNS. This overview highlights recent findings on the alteration of OEM thickness in the adult CNS, explores the underlying mechanisms, and proposes that progressive thinning of OEM represents a novel, nondestructive form of myelin loss in myelin disorders of the CNS.

## Introduction

Myelin is a multilayered structure formed by the extended plasma membrane of oligodendrocytes in the central nervous system (CNS) (Aggarwal et al., 2011; Baumann and Pham-Dinh, 2001; Stadelmann et al., 2019). It wraps concentrically around axons, creating sheaths composed primarily of lipids (70–85%) and proteins (15–30%), which together provide electrical insulation. The lipid components, including cholesterol, phospholipids, and glycolipids, give myelin its insulating properties, while proteins like myelin basic protein (MBP) and proteolipid protein (PLP) stabilize and compact the layers. PLP also shunts cholesterol to the myelin compartment (Werner et al., 2013). Myelin sheaths are segmented into internodes, which are the tightly compacted regions of myelin along axons. These are separated by the Nodes of Ranvier, specialized regions of axons enriched with voltage-gated ion channels. This structural organization allows for saltatory conduction, where action potentials are regenerated exclusively at the Nodes, significantly increasing signal propagation speed while reducing the energy demands of neuronal activity (Aggarwal et al., 2011; Baumann and Pham-Dinh, 2001; Stadelmann et al., 2019). Myelin plays a critical role in ensuring precise synchronization of action potentials by allowing rapid and efficient signal transmission along axons. This synchronization integrates various excitatory and inhibitory inputs, enabling accurate timing of neuronal communication. By maintaining the speed and fidelity of action potentials, myelin supports the coordination of complex neural circuits, which is essential for proper neural network function and processes such as sensory perception, motor control, and cognition. Small alterations of myelin structure can promote or disrupt the synchronization of action potentials and thus influence neural circuit function (Bonetto et al., 2021; Monje, 2018; Xin and Chan, 2020).

A single oligodendrocyte can extend its processes to wrap around multiple axons, forming several myelin internodes (Aggarwal et al., 2011; Baumann and Pham-Dinh, 2001; Stadelmann et al., 2019). Oligodendrocytes also provide metabolic support to axons (Nave et al., 2023). Oligodendrocytes arise from oligodendrocyte progenitor cells (OPCs), a population of resident progenitor cells. During development, OPCs differentiate into mature oligodendrocytes through a tightly regulated process, which can be divided into four stages: OPCs, postmitotic premyelinating oligodendrocytes, actively myelinating oligodendrocytes during myelination, and fully mature oligodendrocytes in adulthood (Baumann and Pham-Dinh, 2001; Emery and Lu, 2015; Marques et al., 2016). In the adult brain, OPCs remain resident and retain the ability to differentiate into mature oligodendrocytes. This allows for the continuous production of new oligodendrocytes and myelin internodes in adulthood, either to replace dying oligodendrocytes or to accumulate new myelin over time (Fernandez-Castaneda and Gaultier, 2016; Clayton and Tesar, 2021).

The thickness of myelin sheaths is a major determinant of the conduction speed of action potentials along axons (Chapman and Hill, 2020; Osso and Hughes, 2024; Xin and Chan, 2020). Traditionally, axon diameter has been regarded as the primary factor influencing myelin thickness, with the assumption that larger axons require thicker myelin sheaths to optimize signal conduction. This relationship is thought to ensure the efficiency of saltatory conduction, particularly over longer distances (Chapman and Hill, 2020; Osso and Hughes, 2024; Xin and Chan, 2020). However, recent research suggests that the link between axon diameter and myelin thickness is more complex than previously understood. Factors such as axon activity, oligodendrocyte-specific signaling pathways, and other regulatory mechanisms also play significant roles in determining myelin thickness (Chapman and Hill, 2020; Osso and Hughes, 2024; Xin and Chan, 2020). On the other hand, evidence suggests that myelination does not dictate the diameter of axons (Bin et al., 2025). For many years, myelin was believed to exhibit remarkable structural stability, including a consistent thickness throughout its lifespan once it was established during early development (Aggarwal et al., 2011; Baumann and Pham-Dinh, 2001; Stadelmann et al., 2019). However, it has become increasingly evident that myelin plasticity is a key component of neuroplasticity, enabling the nervous system to adapt to an ever-changing environment. Emerging studies indicate that the thickness of originally-existing, mature myelin (OEM) can undergo dynamic changes in the adult CNS, contributing to the broader framework of myelin plasticity (Chapman and Hill, 2020; Osso and Hughes, 2024; Xin and Chan, 2020).

Herein, we summarize current knowledge on the mechanisms that regulate the thickness of OEM in the CNS in adulthood, specifically during early and middle adulthood, excluding late adulthood or aging. Moreover, we propose that progressive thinning of OEM represents a new, nondestructive form of myelin loss in myelin disorders of the CNS.

### Neuronal activity alters the thickness of OEM in the adult CNS

The thickness of OEM in young adult rodents can be altered by neuronal activity and behavioral experiences (Chapman and Hill, 2020; Osso and Hughes, 2024; Xin and Chan, 2020). Studies have demonstrated that increasing neuronal activity, either through optogenetic or chemogenetic methods, leads to thicker myelin. For instance, prolonged optogenetic stimulation of cortical layer V projection neurons expressing the excitatory opsin ChR2 significantly enhances myelin thickness (Geraghty et al., 2019). Similarly, sustained chemogenetic activation using the excitatory modified G-protein-coupled receptor hM3Dq results in thicker myelin, reflected by a decreased average g-ratio. Notably, this increase in myelin thickness occurs specifically around axons exhibiting elevated activity (Mitew et al., 2018). In animal models of generalized epilepsy with absence seizures, myelin thickening is observed only after seizure onset, and this effect can be prevented by pharmacologically inhibiting seizures (Knowles et al., 2022). Conversely, social isolation in adult mice leads to reduced myelin thickness in the medial prefrontal cortex, accompanied by decreased expression of myelin-associated genes (Bonnefil et al., 2019; Liu et al., 2012, 2016). Hearing deprivation in adult mice results in thinner myelin sheaths in the trapezoid body, although axon diameter remains unchanged (Sinclair et al., 2017). However, these studies do not conclusively demonstrate that changes in myelin thickness are driven by alterations to OEM sheaths through the addition or reduction of wraps. The potential contribution of newly-generated oligodendrocytes and their associated myelin sheaths to these changes in myelin thickness cannot be ruled out.

### The AKT/mTOR and MAPK/ERK pathways regulate the thickness of OEM in the adult CNS

The AKT/mTOR signaling pathway is involved in regulating various aspects of oligodendrocyte development, including OPC proliferation, migration, survival, differentiation, and myelination during development (Gaesser and Fyffe-Maricich, 2016; Norrmén and Suter, 2013). It has been shown that mTORC1 promotes myelination, but mTORC2 has a minimal effect on myelination in the CNS (Bercury et al., 2014). The critical role of the AKT/mTOR signaling pathway in regulating the thickness of OEM is demonstrated in mice with constitutively active AKT in oligodendrocytes (*PLP/AKT-DD* mice) (Flores et al., 2008). *PLP/AKT-DD* mice exhibit hypermyelination in the CNS, characterized by thicker myelin sheaths, elevated myelin protein expression, and increased mTORC1 activity (Flores et al., 2008). As these mice age, myelin thickness and myelin protein expression continue to increase, eventually reaching pathogenic levels (Flores et al., 2008). Notably, treatment with rapamycin, an mTOR inhibitor, prevents the progressive myelin thickening in *PLP/AKT-DD* mice, indicating that mTOR acts as a downstream effector of AKT in regulating myelin growth (Narayanan et al., 2009).

Previous studies have demonstrated the critical role of the MAPK/ERK pathway in regulating myelin thickness independently of oligodendrocyte differentiation during development (Gaesser and Fyffe-Maricich, 2016; Ishii et al., 2012). Importantly, genetic gain- and loss-of-function studies show that the MAPK/ERK pathway in oligodendrocytes is also a major player in regulating the thickness of OEM in adults. Conditional ablation of ERK1/2 specifically in mature oligodendrocytes of adult mice results in reduced expression of myelin-associated genes and thinner myelin sheaths in the CNS. While some axons become demyelinated, the majority of axons remain myelinated, though with thinner, structurally normal sheaths (Ishii et al., 2014). Conversely, conditional activation of ERK1/2 specifically in mature oligodendrocytes of adult mice leads to increased expression of myelin-associated genes and increased myelin thickness in the CNS, and results in faster conduction velocity and improved hippocampal-dependent emotional learning (Jeffries et al., 2016). Similarly, conditional expression of constitutively active Mek1 specifically in mature oligodendrocytes of adult mice leads to increased ERK1/2 activity, increased expression of myelin-associated genes, and increased myelin thickness in the CNS (Ishii et al., 2016).

### Perinodal astrocytes regulate myelin thickness and conduction velocity in the adult CNS

Myelin sheaths attach to axons by forming a spiral junction in the paranodal region adjacent to the Nodes of Ranvier. In this region, paranodal loops are anchored to axons through septate-like junctions. These junctions are established by a trio of intercellular proteins: neurofascin155 (NF155) on the paranodal loops and the Caspr1/Contactin1 complex on axons (Charles et al., 2002; Dutta et al., 2018). Perinodal astrocytes release thrombin protease inhibitors via vesicles, preventing thrombin-dependent proteolysis of NF155 (Dutta et al., 2018; Dutta and Fields, 2021). Enforced expression of a dominant-negative fragment of VAMP2 specifically in astrocytes of adult mice reduces exocytosis by 50%, thereby promoting thrombin-mediated cleavage of NF155 (Dutta et al., 2018). This leads to detachment of adjacent paranodal loops from axons, elongation of the nodal gap, and thinning of myelin sheaths in the optic nerve, which are accompanied by reduced axonal conduction velocity and impaired visual acuity in adult mice (Dutta et al., 2018). Treatment with the thrombin inhibitor Fondaparinux restores paranodal loop attachment, reduces the nodal gap length, reverses myelin thinning, improves axonal conduction velocity, and rescues visual acuity in adult mice expressing the dominant-negative VAMP2 fragment in astrocytes (Dutta et al., 2018). These findings highlight the critical role of astrocytes in regulating OEM thickness and axonal conduction velocity in the adult CNS. Additionally, evidence suggests that astrocytes can transfer cholesterol horizontally to oligodendrocytes, which may be involved in regulating myelin thickness (Werkman et al., 2021).

### PERK activation in mature oligodendrocytes leads to progressive myelin thinning in the adult CNS

The endoplasmic reticulum (ER), a subcellular organelle, is responsible for the production of myelin lipids and proteins in oligodendrocytes (Lin and Popko, 2009). The unfolded protein response (UPR) and ER-associated degradation (ERAD) are the major players in maintaining ER homeostasis in oligodendrocytes (Hwang and Qi, 2018; Wu and Lin, 2024). Upon ER stress, activation of the PERK branch of the UPR restores ER homeostasis by inhibiting global protein translation through phosphorylation of eIF2α. Recent research highlights the crucial role of the UPR and ERAD in preserving the viability and myelinating function of mature oligodendrocytes in adults (Stone et al., 2020; Wu et al., 2020; Wu and Lin, 2023, 2024). A study utilizing a continuous Sel1L knockout mouse model (*CNP/Cre*; *Sel1L^loxP/loxP^* mice) demonstrates that Sel1L inactivation specifically in oligodendrocytes impairs ERAD, activates the PERK and ATF6 branches of the UPR, and reduces myelin protein translation (Wu et al., 2020). Notably, inactivation of Sel1L in oligodendrocytes does not affect their differentiation or myelination during development but leads to adult-onset, progressive thinning of myelin sheaths and eventually complete loss of myelin sheaths in the CNS, which is accompanied by an adult-onset, progressive tremoring phenotype and eventual death of mice. Notably, this study confirms the absence of demyelination or remyelination in the adult CNS of *CNP/Cre*; *Sel1L^loxP/loxP^* mice, as evidenced by no signs of myelin breakdown or oligodendrocyte death and no increase in oligodendrocyte regeneration (Wu et al., 2020). These findings are further validated using an inducible Sel1L knockout mouse model (*PLP/CreER^T^*; *Sel1L^loxP/loxP^* mice). In this model, Sel1L deletion specifically in mature oligodendrocytes of adult mice similarly results in impaired ERAD, activation of the PERK and ATF6 branches of the UPR, reduced myelin protein translation, and progressive thinning of myelin sheaths in the adult CNS, without evidence of demyelination or remyelination (Wu and Lin, 2023). Furthermore, deleting PERK in oligodendrocytes restores myelin protein translation and reverses the adult-onset progressive myelin thinning in *CNP/Cre*; *Sel1L^loxP/loxP^* mice (Wu et al., 2020). Collectively, these studies suggest that PERK activation in oligodendrocytes induced by Sel11L inactivation leads to progressive thinning of OEM in the adult CNS by inhibiting myelin protein translation.

Furthermore, we investigated the critical role of PERK activation in mature oligodendrocytes in regulating the thickness of OEM in the adult CNS using *PLP/Fv2E-PERK* transgenic mice (Wu and Lin, 2024). These mice express Fv2E-PERK, an artificial PERK derivative whose activity is controlled by a chemical compound AP20187 instead of ER stress, specifically in oligodendrocytes (Lin et al., 2013). To activate Fv2E-PERK in mature oligodendrocytes, adult *PLP/Fv2E-PERK* mice are treated daily with AP20187 or vehicle control for 3 weeks. AP20187 treatment causes activation of the PERK-eIF2α pathway in oligodendrocytes by activating Fv2E-PERK and leads to impaired myelin protein translation and thinning of myelin sheath in the CNS of *PLP/Fv2E-PERK* mice, without affecting the percentage of myelinated axons, axon diameters, or oligodendrocyte numbers (Wu and Lin, 2024). These results provide direct evidence that PERK activation alone in mature oligodendrocytes can result in thinning of OEM in the adult CNS, primarily by suppressing myelin protein translation.

### PERK activation in mature oligodendrocytes leads to OEM thinning in EAE lesions

Multiple sclerosis (MS) and its animal model experimental autoimmune encephalomyelitis (EAE) are inflammatory demyelinating diseases of the CNS. The current conventional wisdoms are: (1) Inflammatory attack causes oligodendrocyte death and myelin breakdown, and then removal of myelin debris by microglia/macrophages results in fully-demyelinated axons (demyelination, a destructive form of myelin loss) in the CNS lesions in MS and EAE; (2) Once inflammation decreases in these diseases, OPCs can proliferate and differentiate into mature oligodendrocytes that remyelinate fully-demyelinated axons in the CNS lesions (Frohman et al., 2006; Bradl and Lassmann, 2010; Lassmann and Bradl, 2017). Activation of the UPR in oligodendrocytes is well documented in MS and EAE (Stone and Lin, 2015; Lin and Stone, 2020). Previous studies have documented that the activation of the PERK and ATF6 branches protects mature oligodendrocytes against inflammation during EAE (Hussien et al., 2014; Lei et al., 2020; Lin et al., 2013; Stone et al., 2018).

**Figure 1:**
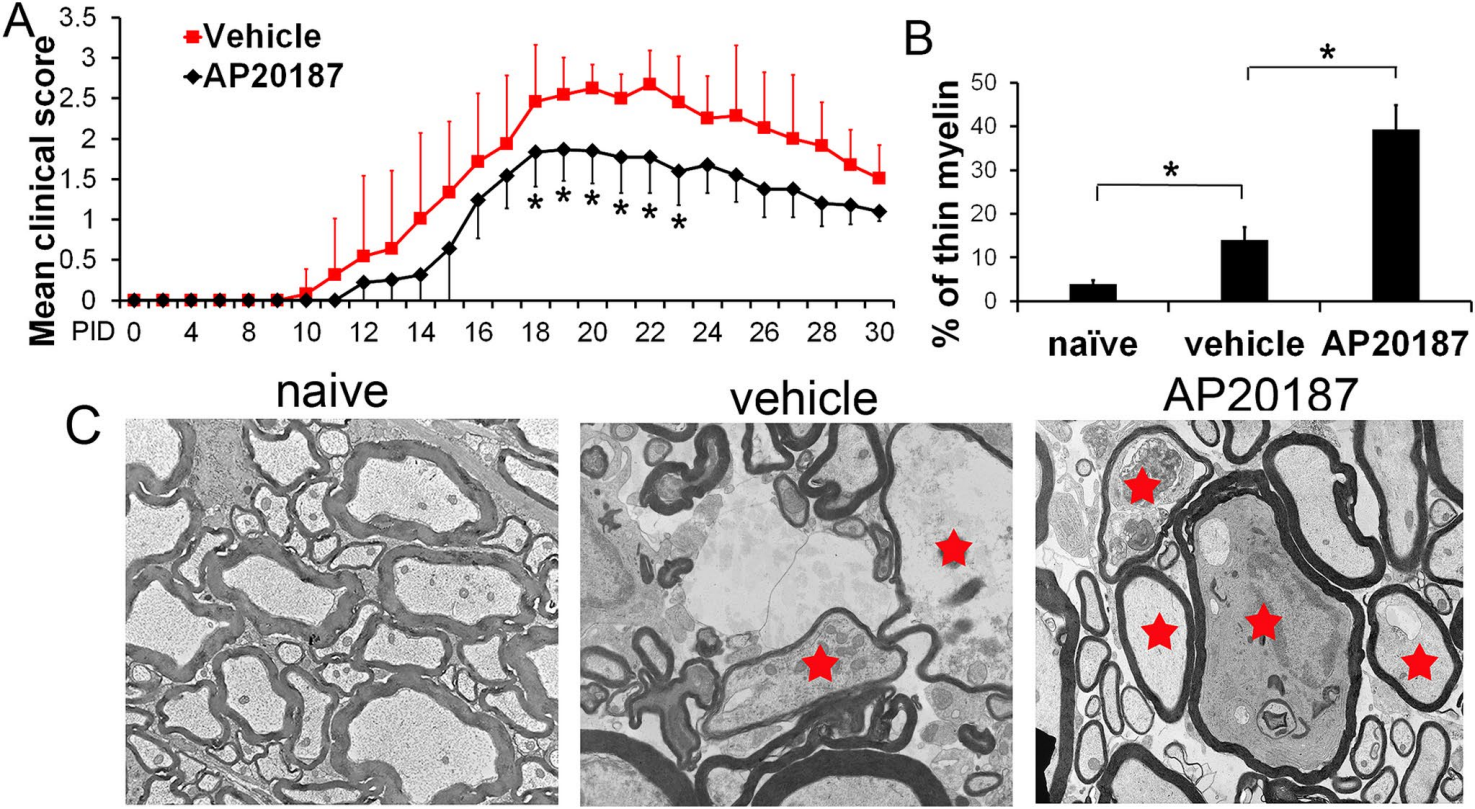
**(A)** Mean EAE clinical score. *PLP/Fv2E-PERK* mice treated with AP20187 demonstrated significantly milder EAE clinical symptoms at the peak of disease (PID18 through PID23) compared to vehicle-treated mice. *N* = 12 mice. **(B,C)** EM analysis showed that the percentage of axons wrapped by thinner myelin (red star) was significantly increased in the lumbar spinal cord of *PLP/Fv2E-PERK* mice treated with vehicle at PID19, as compared to naïve mice, and was further increased in *PLP/Fv2E-PERK* mice treated with AP20187. *N* = 3 mice. Error bars represent SD, *p < 0.05. This was a new representation obtained from analysis of the data published in Lin et al. (2013).

Adult female *PLP/Fv2E-PERK* mice were immunized with MOG peptide 35–55 to induce EAE and then treated with AP20187 or vehicle daily starting at post-immunization day (PID) 10 (before the onset of disease). It has been shown that AP20187 treatment enhances activation of the PERK-eIF2α pathway selectively in oligodendrocytes by activating Fv2E-PERK, resulting in reduced disease severity (Figure 1A), decreased oligodendrocyte death, and diminished demyelination in *PLP/Fv2E-PERK* mice during EAE, as compared to mice treated with vehicle (Lin et al., 2013). We further tested the possibility that PERK activation in mature oligodendrocytes leads to OEM thinning in EAE lesions. Electron microscopy (EM) analysis showed that there were a significantly increased number of axons wrapped by moderately thinner myelin (g-ratio > 0.84) (Figures 1B,C) and the increased numbers of axons that are naked (fully-demyelinated) or wrapped by damaged myelin (Lin et al., 2013) in EAE lesions in the lumbar spinal cord of *PLP/Fv2E-PERK* mice treated with vehicle at PID19 (at the peak of disease), as compared to naïve mice. Intriguingly, AP20187 treatment significantly increased the number of axons wrapped by moderately thinner myelin (Figures 1B,C) but decreased the numbers of axons that are naked or wrapped by damaged myelin (Lin et al., 2013) in EAE lesions of *PLP/Fv2E-PERK* mice at PID19. Moreover, BrdU pulse-chase analysis showed minimal oligodendrocyte regeneration and the minimal impact of enhanced PERK activation in oligodendrocytes on their regeneration in EAE lesions until PID19 (Lin et al., 2013). These results showed that moderately thinner myelin appeared in EAE lesions prior to the appearance of newly-generated oligodendrocytes. The current conventional wisdom is that thinner myelin observed in the CNS lesions in MS and EAE results from remyelination (Duncan et al., 2017; Franklin and Ffrench-Constant, 2017). Because newly-generated oligodendrocytes are essential for remyelination (Franklin and Ffrench-Constant, 2017), this finding suggests that moderately thinner myelin in early EAE lesions results from OEM thinning rather than remyelination. These results also showed that enhanced PERK activation in mature oligodendrocytes decreased the number of axons that are wrapped by damaged myelin or fully-demyelinated, increased the number of axons wrapped by moderately thinner myelin, and did not alter oligodendrocyte regeneration in early EAE lesions, suggesting that PERK activation in mature oligodendrocyte prevents demyelination (a destructive form of myelin loss) but induces OEM thinning (a nondestructive form of myelin loss) in EAE lesions. However, due to the absence of a technique capable of longitudinally monitoring OEM thickness in EAE lesions, it remains unclear whether thinning of OEM ultimately results in complete loss of OEM sheaths in later EAE lesions, warranting further investigation.

### Concluding remarks and future perspectives

Recent literature explores alterations in OEM thickness in the adult CNS, shedding light on its underlying mechanisms. Myelin loss occurs during myelin remodeling and replacement as well as in various neurological diseases, including demyelinating and neurodegenerative conditions (Huang et al., 2024; Stadelmann et al., 2019). While demyelination—a destructive form of myelin loss—is well documented under these conditions, recent studies suggest progressive thinning of OEM, a nondestructive form of myelin loss, in both physiological and pathological states. Notably, research indicates that activation of the PERK pathway in mature oligodendrocytes can drive this progressive thinning of OEM. Activation of the UPR is well documented in oligodendrocytes across various myelin disorders and neurodegenerative diseases (Hetz and Saxena, 2017; Lin and Popko, 2009; Lin and Stone, 2020). Therefore, there is a possibility that progressive thinning of OEM caused by PERK activation in mature oligodendrocytes represents a new, nondestructive form of myelin loss in various myelin disorders and neurodegenerative diseases. However, current evidence supporting dynamic changes in OEM thickness relies heavily on EM. Although EM is the gold standard for assessing myelin thickness, EM is limited by its inability to longitudinally monitor myelin dynamics. Developing technologies that allow longitudinal assessment of myelin thickness is urgently needed to validate the dynamic change of OEM thickness in the adult CNS and the occurrence of progressive OEM thinning (a new, nondestructive form of myelin loss) in myelin disorders and neurodegenerative diseases. On the other hand, the widely accepted biomarker for differentiating newly-generated myelin from OEM in myelin disorders is based on the assumption that newly-generated myelin is thinner than OEM (Duncan et al., 2017; Franklin and Ffrench-Constant, 2017). Nevertheless, identifying progressive thinning of OEM challenges the dogma that thinner myelin is a reliable biomarker for remyelination in myelin disorders.

## Data Availability

The original contributions presented in the study are included in the article/supplementary material, further inquiries can be directed to the corresponding author.
